# Copper as a Preventive of Cholera

**Published:** 1870-08

**Authors:** 


Copper as a Preventive of Cholera.—M. Berg says a society of coppersmiths, through four successive cholera epidemics, enjoyed almost complete immunity from the scourge, while people of all classes around them were dying by hundreds. From minute inquiry he learned that the mortality among copper workmen was only five in 100,000. The immunity seems to be in proportion to the relative amount of fine copper (copper powder) which the men can inhale.—"*Jour. de Med., &c.,*" *Ibid.*

